# Open drug scenes: responses of five European cities

**DOI:** 10.1186/1471-2458-14-853

**Published:** 2014-08-16

**Authors:** Helge Waal, Thomas Clausen, Linn Gjersing, Michael Gossop

**Affiliations:** Norwegian Centre for Addiction Research (SERAF), University of Oslo, Oslo, Norway; Clinic for Psychiatry and Addiction, Oslo University Hospital, Kirkevn 166, 0450 Oslo, Norway; Norwegian Institute of Alcohol and Drug Research, Oslo, Norway; Kings College London, National Addiction Centre, London, UK

**Keywords:** Drug use area, Public nuisance, Harm reduction, Urban drug policy

## Abstract

**Background:**

Open drug scenes are gatherings of drug users who publicly consume and deal drugs. The authors conducted a study of five European cities that have met such scenes constructively. The aim was to investigate shared and non-shared interventions and strategies in order to increase the understanding of this type of problem.

**Methods:**

First a description was made for the cities of Amsterdam, Frankfurt, Vienna, Zürich and Lisbon. These descriptions were sent to contact persons in each city prior to visit by the researchers. The methods and strategies in each city were discussed and core choices and efforts extracted. A report was sent to the contact group for corrections and clarifications. The paper analyses shared and non-shared traits and strategies.

**Results:**

All of the cities had initially a period with conflict between liberal and restrictive policies. A political consensus seems to be a prerequisite for effective action. A core shared characteristic was that drug dependence was met as a health problem and drug use behaviour as a public nuisance problem. Low threshold health services including opioid maintenance treatment were combined with outreach social work and effective policing.

**Conclusion:**

Prevention, harm reduction and treatment should be combined with law enforcement based on cooperation between police, health care and social services. The aim should be “coexistence” between society and users of illegal substances and the strategy based on several years planning and conjoint efforts. The solutions are found in appropriate combinations of harm reduction and restrictive measures.

## Background

When drug users congregate to sell and use drugs in public spaces, this phenomenon is referred to as an open drug scene [1]. The phenomenon was studied in a cross-national project in the early 1990’s that focussed on nine European cities. The study was financed by European Monitoring Centre for Drugs and Drug Addiction (EMCDDA) [2]. According to the authors there was neither a generally accepted definition of a “drug scene” nor an agreement on what was meant by “open”. The study therefore developed an operational concept defining open drug scenes as all situations where citizens are publicly confronted with drug use and drug dealing [2]. These scenes vary in visibility, size and site and might be categorized as concentrated open scenes, dispersed open scenes, and hidden scenes. The advantages and disadvantages of dispersed drug use areas versus concentrated scenes have been discussed [3, 4]. Some observers have advocated that the concentrated scenes might ease control and monitoring of an illegal drug market. The effectiveness of harm reduction measures such as outreach services, dispersion of sterile utensils and location of user rooms might also be increased as they could be provided in the same area [5–8]. However, low drug prices, lack of law enforcement and lack of social control seem to attract drug users towards the open drug scene and the increase in problems appears to have been more rapid than the increase in the population of addicts [9].

This paper focuses upon the concentrated open scenes where users meet and use drugs in ways that are visible to the public. The scenes are often found in city centres, particularly in central railway stations and city park areas. The users may be in poor physical and mental condition and the locations will often serve as a focal point for drug dealing. This situation might grow out of police control as has previously happened in European cities such as Zurich and Frankfurt [1, 2, 9, 10]. Also, the scenes are often feared to attract new users and antisocial groups and individuals. The stigma and negative image seem mostly to discourage well integrated adolescents from approaching the scenes [11], but a mixture of push and pull-factors might attract less socially stable adolescents [12, 13], displaced persons, asylum seekers and others who have problems fitting into the societal structures. Homelessness is often prevalent as different types of dysfunctions are prevalent [14, 15] and increased rates of crime may be found in the neighbourhood. For such reasons, the scenes are regarded as a public nuisance and the usual consensus is that an open concentrated drugs scene is a problematic phenomenon. However, there have been different views on how to reduce or prevent the drug scenes. In their study Bless and colleagues [2] state that the cities adhered differently to preventive and corrective strategies often influenced by ideological concepts where “prohibitionists” confronted adherents of “harm reduction”. Closure by repressive measures might increase drug related difficulties [1, 16, 17], but although harm reduction measures might decrease the social and health harms [8], they do not seem to decrease the size and seriousness of the open drugs scenes. Closure might increase the propensity of users stuck in the scenes to seek treatment noted as increased demand for methadone maintenance [18] but might also increase user desperation and destructive patterns of drug use. These experiences are relevant for discussions on drug policies.

Open drug scenes have been described in cities such as Vancouver and Montreal in Canada, New York and San Francisco in the US, and Amsterdam, Rotterdam, Frankfurt, Hamburg, Zürich, Vienna and Copenhagen and Oslo in Europe. In 2004 the Pompidou group launched a series of seminars to study drug scenes and the different national policies and prepared a list of recommendations [19]. With this as background the authors performed a study of the interventions applied in European cities known for successful reduction of open drug scenes. The aim was to investigate the core interventions and strategies and describe the evidence for effectiveness. The study was commissioned as a part of planning preventive interventions to address the problem of an open drug scene in Oslo in 2009.

## Methods

This study built on earlier involvement by the first author with a seminar in the Pompidou group on urban security [20]. An interdisciplinary study group was established; in addition to researchers this included persons with expertise on addiction, urban city policies and police strategies; a specialist in psychiatry and addiction medicine, the head of the clinical substance user services in Oslo, a high ranking police officer and two researchers. Five cities that were well known for their responses to open drug scenes, were selected; Amsterdam, Frankfurt, Zürich, Vienna and Lisbon. As the study of interventions in different social setting does not lend itself to experimental or controlled investigation, a method combining qualitative and quantitative observations was chosen in accordance with the Qualitative research review guidelines – RATS [21].

The report to the Pompidou group was supplemented with national reports from the EMCDDA on the drug problems in selected countries. In addition an unsystematic literature search was performed in Medline, Pub Med, Psychlit and Sociological Abstracts with the search words “open drug scenes”. As the period studied stretched from changes in Amsterdam in late 1970’s to changes in Vienna and Lisbon up to the time of the study, the search had no pre-specified time limit. The first author selected the papers found relevant on basis of abstract before reading in full text. All papers dealing with description of drugs scenes in the core cities and the measures taken in the cities were included. Papers primarily focussing HIV or HCV prevention in general were excluded as were papers focussing trials with heroin assisted treatment. Papers focussing aspects such as gender problems on the scenes and other cultural aspects were likewise found outside the scope of the study. The available evidence describing the development in each city was concentrated to specific city descriptions focussing upon the characteristics of the open drug scenes and the city responses and core strategies. The information was discussed and organized in descriptions according to the groups knowledge of the cities. These descriptions are based on consensus in the group.

A public office or authority with coordinating or planning responsibilities in the cities was then approached with a request for a contact person. The city descriptions were sent to the contact person in each city for input and corrections. The contact persons arranged for meetings between the visiting group and the core city planning office, addiction treatment experts and core police officers in order to have discussions on relevance and importance of chosen methods. In addition the group visited the former open drug scenes. The cities’ interventions and the visiting group’s understanding were discussed in meetings with researchers, police, social workers and addiction medicine specialists. The resultant observations are therefore based on a consensus developed from different sources.

On this basis, a description and analysis of the measures and strategies were prepared for each city. The analyses were discussed in the group and then sent to the contact person in each city for final comments. This final revision serves as the basis for findings of shared and non-shared experiences and core interventions in this paper. Each city was visited for 2–3 days. As we were dependent on meetings with professionals that mostly have heavy workloads and busy schedules, the meetings were adjusted to their possibilities. Each meeting lasted from one to two hours. The main focus were on the preformed city descriptions, the correctness and relevance of interventions described and relative importance of the interventions and the experiences. The agenda was open to allow for the focus and judgement of the partaking professionals both from the visiting group and the city representatives. More detailed descriptions of the development of drug problems in the cities, the chosen contact persons, details of the visits and on relevant socioeconomic city characteristics can be found in the report to the municipality of Oslo [22].

### Ethics

Health research in Norway is regulated through the Act on medical and health research that covers research that include health indicators or personal information of any type concerning individuals or treatment that can be traced back to individuals. Studies on general treatment quality and on effectiveness of economic or other regulations are outside the scope of the statute that regulate applications to ethical boards [23]. As the study concerns city policies on the general phenomenon of open drug scenes, application to research ethical board was judged unnecessary.

## Results

### Amsterdam - “the introduction of harm reduction”

Description was based on Bless [2], Bless [24], Buning &van Brussel [25], Buster [26], Kalmthout [27], Reinaas [10], Waal [20], van der Meer [28], EMCDDA national report [29].

### Open drug scenes

Problems in Amsterdam were first evident with the development from cannabis use integrated in an anti-establishment culture towards increasing use of heroin among disadvantaged groups. Initially in the late 1970’s these problems were met with measures of prevention and repression. In spite of these measures, the number of drug users in the city with problematic and self-destructive behaviours increased. Open drugs scenes appeared early. A well-known example is the development of the problem in the Zeedijk area, a deprived city area with slum characteristics.

### Responses

After initial discussions and shifts in policy, the Amsterdam City Council asked the Amsterdam Municipal Health Service to develop strategies to reach the “unmotivated drug users”. The aim was a public health approach both to contain the “drug epidemic” and to meet the specific needs of this group. This can be seen as the origins of harm reduction as a systematic policy. One characteristic feature was the attempt to separate “soft drugs” (i.e. cannabis) from “hard drugs” (in particular heroin) [25, 27]. Drug use was not seen as a crime while professional dealing was. Dependence was regarded as a disease to be met by health care measures.

Another feature was heavy investment in low threshold methadone dispensing from the Municipal Health Service [25]. Mobile dispensing from buses was started in order to reach marginalised groups and to overcome resistance from unwilling neighbourhoods. Methadone dispensing from police stations was initiated to reach deviant and antisocial groups and to enable continuation of treatment after arrest and imprisonment. Needle dispensing was also initiated, as were shelters and contact centres. The main purpose was a systematic policy of harm reduction and health care policies. After the turn of the century this was supplemented with heroin assisted treatment, compulsory treatment within mental health systems and quasi-compulsory treatment in special prisons.

### Core strategies

Open drug scenes were, from the beginning, systematically met with policing and an extensive redevelopment programme in affected areas [2]. As the drug scenes became more prominent, the policy emphasis increasingly focused on dispersion of scenes, urban safety programmes and compulsory interventions aimed at street addicts who were not responding to helping measures. Any public gathering of more than four to five addicts was to be interrupted by the police backed by administrative laws that authorised fines. If the users did not pay their fines, this could result in the issuing of court orders followed by arrests. The courts could also impose antisocial behaviour orders.

The cultural attitude of tolerance towards deviant behaviour is also relevant. The Dutch tradition seems to contain a high tolerance for self-determination provided there is no public nuisance. The police have traditions for making of alliances with deviant groups and to find compromises so that the law is practised leniently or adapted to situations where non-action might be sensible.

### Observations

Bless [2] noted that the Amsterdam experience suggests that a consistent approach along these lines can be quite effective to keep the scene on the move and prevent major concentration of drug users. On one hand, treatment and harm reduction centres are readily accessible. On the other hand, drug users that cause nuisance might be subjected to compulsory means, including choice between prison and treatment. The system is presently reported to meet with broad satisfaction by the community.

### Present situation

At the time of the present study, there was no noticeable open drug scene. The combination of a harm reduction strategy and systematic prevention of public nuisance had been effective in keeping the problems to tolerable levels. Open scenes were present but only in a dispersed form and not publicly noticeable. The main current emphasis was to develop the heroin assisted treatment programmes, to increase integration with the psychiatric treatment system, particularly with assertive community teams, to strengthen comprehensive treatment for those with co-morbid problems, to strengthen treatment within prisons, and, more generally, to integrate health system based addiction services, social service systems and mental health systems.

### Frankfurt Am Main -”taunusanlage and user rooms”

City description was based on Bless [2], Hedrich [7], Kemmesies [17, 30], Reinaas [10], Waal [20], EMCDDA national report 2009 [31], Schard [32].

### Open drug scenes

Problems in Frankfurt began with cannabis smoking and gatherings in parks. These were initially met with abstinence-oriented treatment and police repression. Such responses were largely ineffective and were followed by increasingly destructive drug use. The latter half of the 1980’s brought development of a large and destructive drug scene in the “Taunusanlage”, a park area between the old and new opera, and with large bank buildings on both sides. The scene grew until more than one thousand users met daily with open dealing and injecting drug use, obvious mental and physical problems, and public nuisance.

### Responses

An initial policy of abstinence-oriented treatment was gradually changed towards harm reduction with contact centres and methadone places, outreach services, and services offering basic medical assistance. Periodically the police would disperse the scenes and arrest users and dealers in an inconsistent policy with shifts between liberal and restrictive measures. This situation was seen as unsatisfactory, and in 1987 the city established a coordinating committee, and in 1989 a city coordination office.

### Core strategies

In 1992 the Mayor decided that the open drug scene in the Taunusanlage could no longer be tolerated. Low threshold methadone programmes were enlarged and decentralized. A large shelter with a contact centre, cafe and a methadone outpatient clinic opened in former industrial buildings outside the city centre. Then a massive police intervention was implemented. Drug users not resident in Frankfurt were expelled from the city. Helping facilities were at the same time established in the home communities. Users in the city centre and at the scenes were taken by bus to the contact centre in the periphery. The first safe injection room was established in 1994 and three more in 1996.

### Observations

According to our contact group the present treatment system is well received both by the public and the political system. Shelters were closed in daytime, the rules for conduct were explicit and exceptions were only accepted in very special cases. The system with a combined reliance upon harm reduction for users and zero tolerance for public nuisance seemed to be integrated in the city. There was a strong emphasis on social integration of users, but also an expectation of compliance. However, the original system of removing non-resident users to their home locations was often ineffective and was easily counteracted by a return ticket to Frankfurt.

### Present situation

The current policy decisions and measures were originally met with opposition and demonstrations, but at the time of the study open drug scenes were not seen as a significant problem by the respondents. Although there has been a continuous establishment of new drug scenes, these have been kept under control by outreach social services in cooperation with city police. There are approximately 200 to 300 users who belong to a drug scene around the central train station in Frankfurt’s red-light district. Here a small group of 30 to 50 persons are “causing trouble and are viewed as unruly and with no respect for the police”. The capacity in the health system and social services is relatively satisfactory and there are no waiting lists in methadone maintenance programmes or shelters. However, capacity for long term rehabilitation is unclear, and there is a high level of homelessness and unemployment. Users who neglect the rules of the shelters are often asked to leave, if necessary with the assistance of the police. Difficult and violent users are subjected to court proceedings according to possible crimes, and might also be handled with in-house sanctions (e.g. problematic individuals may be denied admission for a time). The mental health system is expected to take care of addicts with psychiatric disorders. The heroin assisted treatment programme in its current form was not structured to treat the most problematic users and was therefore not relevant to most of the users in the open drug scenes.

In general, the situation was reported to be acceptable, and it was intended that the policy should continue along the established lines. Respondents stated that a certain level of problems should be expected and integrated in a city the size of Frankfurt. The need for a long term perspective was especially emphasized with no quick solutions. Several years’ perspective is necessary to deal with open drug scenes with continuing efforts to prevent recurrence. Coordination of control and harm reduction sectors is a core element as is continued focus on problem solving and monitoring.

### Zürich - the city of heroin treatment

City description was based on Bless [2], Uchtenhagen [33–35], Klingemann [36], Falcato [18], Fuchs [5], Huber [9], Stohler [37, 38], Hertzig [39], Conolly [19], Waal [20].

### Open drug scene

Users began to congregate in parks and open places during the late 1970’s and the problems increased through the next decade. Repressive measures and helping measures were both advocated and tried. The situation was characterized by political controversies and shifting decisions. Finally a large open drug scene developed in the “Platzspitze” (needle-park) in central Zurich. Several thousand drug users gathered daily, selling and openly injecting drugs. Many supportive services were provided at the scene and these helped to alleviate some of the problems, but the scene expanded regardless. A massive closure initiative merely caused the scene to move to a nearby unused railway station, the Letten, with a subsequent increase in problems.

### Responses

The situation in the early 1990’s was experienced as catastrophic. In response to this situation the leading political parties developed a shared policy platform, and this enabled the national authorities to develop a national plan with four pillars; prevention, treatment, controls, and harm reduction. The plan was based on coordination at the municipal, canton and national levels, with the cooperation of police, treatment system and social authorities. Outreach services cooperated with the police and interventions could include police detaining users against their will. Methadone treatment provision was massively enlarged and later supplemented with heroin assisted treatment. Harm reduction was promoted as the primary aim with treatments adapted to the needs of the user. Nobody was excluded for drug use or non-compliance. However, retention was often limited, with many users leaving treatment though many might return again at a later stage. Also, users who continued public nuisance behaviour might be brought to “relocation centres”, possibly leading to quasi-compulsory treatment. Violent users were judged to be a shared responsibility for control and treatment sector. Mentally ill users were regarded as the responsibility of the public mental health system with well-developed traditions for opioid maintenance treatment.

### Core strategies

The Platzspitze was closed in 1992, and the Letten scene in 1994. All users were approached with offers of treatment, and these were linked to control measures and repression of the drug scenes with users eventually compelled to return to their home municipalities. As open drug scenes tend to recur, a continuous joint effort was established. Our respondents emphasized that the police initially defined the drug scenes as social problems while the social workers tended to view the scenes as a public order problem. What was needed, according Zürich respondents was a shared understanding of a joint responsibility. This is best developed through daily meetings between police, social and health care workers. A core concept is “Urban compatibility” (Stadtverträglichkeit) with the aim to integrate marginalized individuals. Homelessness is defined as unacceptable, and Zürich has 1500 housing places for different target groups, about hundred places in temporary shelters and about 400 low threshold places for drug users and other socially marginalized individuals. There is z*ero tolerance* for certain types of behaviour such as drug dealing and for large gatherings of users which is seen as “destructive to co-existence” in public places. In addition Zürich has created a specific approach termed “SIP” (Security, intervention, prevention). This involves outreach social services working in close cooperation with the police with shared information systems. The intention is to educate marginalized people to social behaviour and the aim is co-existence.

### Observations

The Zürich situation may be understood in terms of responses to a national and local crisis with an unprecedented rapid increase in heroin use, large open drugs scenes and related criminality and mortality. This crisis seems to have ended several years of conflict between liberal and conservative parties advocating different measures with treatment and survival measures versus repressive control measures. Repeated shifts of policy were replaced by a long-term systematic policy integrating prevention, harm reduction, treatment and control. During our discussions it was repeatedly emphasized that all pillars have equal importance. The integration of control measures with treatment and harm reduction was especially prominent. Another important aspect was the overall coordination of national, cantonal, and municipal policies. Today the policies have broad general acceptance in the general population and the political parties accept and support the policies included the different harm reduction aspects, with exception of a right wing extremist party.

### Present situation

Open drug scenes have now largely disappeared in Swiss cities. Drug dealing typically occurs in private apartments. Drug injecting in public places is infrequent. Used needles and syringes in public places are reported to be a minor problem. Also, user satisfaction is reported to be high, particularly in projects using heroin assisted treatment. Control measures are reported as being integrated with treatment, health care and social services. The capacity of health and social services is reported to be sufficient with well-developed systems of quality control. Involuntary and quasi compulsory treatment is accepted. Treatment in prisons is well developed with differing models.

### Vienna- ”zusammenleben” and “zones of tolerance”

City description was based on Bless [2, 24], Springer [40], Uhl [41], Waal [20], EMCDDA annual report Austria [42].

### Open drug scene

A growing drug problem during the late 1980s increased greatly during the nineties. Open drug scenes and their associated features caused heated public debate. The result was a high level political decision to appoint a drug coordinator and a coordinating body. A policy of “zusammenleben” was formulated to contain the problems and reduce the harms and nuisances. The basic policy element was to confront user areas with diversion and “zones of tolerance”. Originally several small satellite drug scenes existed. But gradually the treatment system was increased and the scenes prevented by repressive measures. The last such zone was in the park at Karlplatz where 40–50 users were tolerated at one time. Roughly 1000 users belonged to the scene. Outside the zone no more than four to five persons were allowed to gather, particularly not in the Metro. If more than 10 users gathered outside the zone, they would be asked by the police to move and to disperse, or to go to the zone. The zone was under police surveillance and outreach social workers worked in the area. Apart from this, there were no other services at the scene: this was the result of a deliberate strategy not to increase the attractiveness of the scene in a precarious balance between too much control and restrictions (repression) and too little (too much tolerance). The main problems with Karlplatz, according to our informants, were the visibility of drugs and intoxication. There were no injections on the scene, but nearby toilets were used as injection rooms. It was also a difficult place to oversee, and included food stalls, shopping malls and a centre for several Metro lines and buses. The social concerns increased.

### Responses

Austria initially developed a strategy of treatment and containment. Methadone maintenance treatment, and subsequently maintenance with slow release morphine was made available both by general practitioners and from public programmes. A publicly funded social organisation was developed to provide social support systems with meeting places and help. Vocational training programmes had been an important part of the city response together with a large housing programme as the city has a large number of public apartments. There was a consensus that addiction is an illness and that users are primarily the responsibility of the health care and social care system. The principle is treatment for addicts and repression for dealers. Users are generally not imprisoned. Maintenance treatment should be available, if necessary on demand with a low threshold. A high emphasis is placed on out-reach and low threshold services.

### Core strategies

From the turn of the century, pressures on smaller open drug scenes increased until Karlplatz remained as the only remaining scene. In 2010 it was decided that this should also be closed. Reconstruction of the bus station offered an opportunity. Social services increased the availability of counselling and shelters, and cooperation between social services and the police was strengthened. The number of staff in the contact centres was increased and two new places for needle exchange established. The capacity of the night shelter was doubled. The contact centre and needle exchange at Karlplatz were moved from the area while the street worker contact group remained. The surrounding park was reconstructed with increased visibility. Then the drug scene was closed down by police interventions. When the study group visited the scene there were almost no drug users present and the closure was viewed as having succeeded. However, it was stated that continuing intervention to prevent the re-establishment of the drug scene was necessary. At least two police officers were always to be in sight and the intention was to prevent the re-emergence of any open scene: this policy was to be continued for at least two to three more years. The goal was to get drug users into the treatment system and to avoid users having to buy drugs illegally on the black market.

### Observations

Most users can presently find treatment or harm reduction measures in a variety of therapeutic settings, counselling centres, GPs, and inpatient treatment. Only a minority of users are outside the treatment system. User satisfaction is reported to be high, but more psychiatric help is needed for co-morbid users. A high level of health and social care is a priority of the system. It is stated as a goal that no drug user should be without a home and the homeless care services are well developed. Nearly 100% of all drug users are covered by the health care system and social insurance. According to the city sources, there are only minor problems with difficult users. Users who sell drugs on premises, use violence against other clients or threaten personnel will be excluded: however, they can find treatment in any other institution. Continuing drug use is not seen as a reason for exclusion from treatment. Violent users are very few and dealt with individually. As a last measure police are called in. It is an intention to engage more psychiatrists in drug institutions and to develop psychiatric treatments specifically to meet the needs of mentally ill users.

### Present situation

The Vienna system seems to be a functioning well without heroin dispensing and injection room facilities. The system offers a high level social and health care system operating on a harm reduction model together with an emphasis on the prevention of public nuisance. The earlier policy of zones of tolerance, rules of conduct and conflict management has been modified and the zones have been closed. The level of overdose mortality is viewed as within acceptable limits even though maintenance drugs appear to be the main opioid involved in these deaths. Open drug scenes are presently prevented through continued police surveillance and outreach social service interventions. A decentralized system of opioid maintenance treatment, largely in primary care settings, with different types of opioid agonists is provided.

### Lisbon – decriminalization as a solution?

Description was based on Bless [24], Greenwald [43], Hughes [44], EMCDDA Annual report Portugal.

### Open drug scenes

Before downfall of the dictatorial regime of Salazar in 1974 the army and the navy had an important place in Portuguese society and large neighbourhoods were dependent on navy activities. The political changes brought hardship, and drug trafficking became a possibility to supplement income. Three large areas/neighbourhoods in Lisbon developed into open drug scenes termed “supermarkets”. Casal Ventoso, the largest “supermarket”, had approximately 5000 “visitors” every day. 2000 people lived in the area and whole families were involved in trafficking. In this area there were large numbers of individuals in poor health and conspicuous deprivation, openly injecting drugs.

### Responses

A general change of policy was formulated by the “Commission for a National Drug Strategy” which regarded drug use as a health problem. Drug use was largely decriminalized, and individuals were permitted to carry user doses for 10 days without charges. Selling remained criminalized. At the same time Portugal established a specific system based on administrative sanctions, the “Commissions for the Dissuasion of Drug Addiction” (CDT). Police and other authorities were able to refer users to these regional bodies that have psychologists, and social workers. The CDT functions as a sort of court, informing the user, listens to them and decides on responses. The user is also entered in a national register separate from the criminal register. This system is intended for non-dependent users. Addicts should be referred to treatment. Civil behaviour orders are also issued. The CDT system should be understood as a part of a comprehensive treatment system that is intended to function without waiting lists and with outreach social services that might support motivation and problem solving among users.

### Core strategies

The emergency responses were developed in collaboration with the municipality. The open drug scene (“supermarket”) areas were literally destroyed and rebuilt with EU grants. At the same time treatment availability was increased and low threshold opioid maintenance treatment services such as methadone buses were established. At present, open drugs scenes are constantly met with a police presence, and the police can refer users to the CDT system. The police have the authority to search users on the street and to control areas with obvious open drug scenes.

### Observations

Initially, these responses prompted a heated debate and strong negative predictions about future increases in problems. However, the city informants stated that the level of problems had diminished, and at the time of the study, drug use was not seen as a major public problem. The realities are difficult to evaluate. It seems clear that the system presently is well accepted. It seems also clear that most users are positive about the CDT system as an alternative to court proceedings. The system is, however, not in any way a form of legalization. On the contrary, in several ways this type of decriminalization might be seen as relatively strict societal response.

### Present situation

The former, large open drug scenes seem to have disappeared. However, drug use in cafes and on the party scene is prevalent, and drug selling, at least of cannabis, is not uncommon in Lisbon. The number of users in treatment has increased while the growth of drug problems has stalled or diminished. The CDT system is mostly respected by users, but repeated use and repeated referrals might increase levels of sanctions. Mental health problems should, at least in theory, be diagnosed and followed up within mental health services, and city informants report that the drug treatment services are part of or work in close cooperation with psychiatry.

### Shared and non-shared interventions and strategies

Table 1 gives an overview of the city interventions and strategies. As can be seen all the cities have shared policy decisions with strategies for coordination of different measures and of communication between control sector and social sector. Further, all have well-known harm reduction interventions although user rooms are only found in three of the cities. Heroin assisted treatment is well developed in two and on a small scale in one. All have well developed system of shelters, and housing is mostly available as is contact centres. The control sector interventions are less shared but all share dispersing tactics. Some form of social behaviour order is found in four out of five. Strategies to discourage or send out non-residents are also usual. Formal decriminalization of substance use is only found in Lisbon while informal is also found in Amsterdam and Zurich as long as it does not occur in public. All cities have zero tolerance for public nuisance (not shown in table).

**Table 1:** **Shared and non-shared interventions and strategies**

	Amsterdam	***Zurich***	Frankfurt	Vienna	Lisbon
Shared political and social consensus	+	+	+	+	+
City coordinating system	+	+	+	+	+
System for shared information	+	+	+	+	+
Low threshold OPIOID MAINTENANCE TREATMENT	+	+	+	+	+
Needle dispensing	+	+	+	+	+
Health rooms/user rooms	+	+	+	-	-
Heroin assisted treatment (HAT)	+	+	(+)	-*	-
Housing projects	+	+	-	+	+
Shelters	+	+	+	+	+
Contact centre and café	+	+	+	+	-
Outreach social work services	+	+	+	+	+
Compulsory treatment	+	(+)	-	-	-
Reconstruction projects	+	-	-	+	+
Shared policing and social services patrolling	+	+	-	+	-
Zones of tolerance	-	-	-	+	-
Detention centre	-	+	-	-	-
Police dispersing tactics (compulsory)	+	+	+	+	+
Social behaviour orders	+	+	+	-	**
Treatment within criminal justice	+	+	-	-	-
Decriminalization of personal use	(−)	(−)	-	-	+
Expulsion/discouragement of non-residents	(+)	+	+	+	-

*Vienna offers maintenance treatment with slow release morphine.

**Commission for Dissuasion of Drug Addiction (CDT).

## Discussion

The cities in this study were chosen primarily because they have been successful reducing open drug scenes. In Amsterdam, Zurich and Lisbon, the city strategy was embedded in national drug policy and strategy reforms but all were in consort with drug policy development. Amsterdam chose early a policy of harm reduction to counteract growing problems, but as the study demonstrates, these are combined with systematic control measures. This seems to be a shared trait. All the cities describe open drug scenes that tended to grow out of control. These scenes were regarded as destructive to individual drug users and as a problematic nuisance to society. Then the cities applied a range of harm reduction measures combined with systematic control strategies in order to tackle the problems. None of the cities succeeded by treatment and medical and social support measures alone. Provision of increased helping measures alone seems to have been unsuccessful and when provided within the open drug scenes, may even have increased their attraction. Equally, the provision of repressive methods alone has not been successful, even when an increase in control measures was combined with crisis intervention and coercive interventions. Only when the cities developed a comprehensive policy that integrated and coordinated treatment and helping measures with control measure were they able to successfully alleviate their situation. It might seem that the all have met the precondition of overcoming the controversy between prohibitionist and harm reduction ideologies as observed to be important by Bless and co-authors [2]. As pointed out by Csete [45] in discussing the case of Zürich, a pragmatic approach shows the usefulness of different harm reduction elements within a comprehensive urban policy. This is also reported from Rotterdam where “tolerance zones” and comprehensive helping measures did not prevent troublesome presence in the streets of homeless and addicts. “Silence in the streets” was attained only when the city developed a “pro-active” policy combining control measures with care for the client [46].

The comprehensive and integrated policies share certain core features. One is that problematic substance use and dependence are defined as health care problems. At the same time, drug taking is seen as a social behaviour that is subject to ordinary social rules. The user has no right to be of nuisance to others, but the user has the same inborn right to integration in society and the same set of individual rights as others in the general population. Social stigmatization and isolation should be avoided. It is also accepted that mental health problems are often a key feature of problematic drug use and that these conditions should be diagnosed and treated effectively. Nevertheless, problematic behaviour should be controlled and prevented, and relevant measures have to be shared across different professions and service systems. Therefore shared responsibility should be accepted with a commitment to cooperation between the police, the social services and the health care services. This commitment has to be binding and anchored at a high political level. A common observation was that where there had been political and professional disagreements and conflicts, these obstructed the implementation of any effective policy. A common theme was that on-going political and ideological conflicts seem to have prevented solutions and effective measures for several years. Only when consensus had been reached at sufficiently high political and administrative levels, has real progress been achieved. Another important aspect was long term policies. The cities all emphasized a time perspective of several years for the implementation of both helping and control measures. There are no quick fixes. In particular; election periods were seen as too short a time perspective, meaning that a consensus between the major political parties is essential.

A conspicuous feature is that harm reduction has been adopted as a central strategy in all the studied cities. There were differences in how this strategy was implemented but the common features were a free-of-charge, low threshold public health service, often at city service level. All the cities established easy access to low threshold opioid maintenance treatment without or with short waiting periods. This is most likely a precondition for closure of drug scenes that up to closure have secured availability of heroin.

Another feature is that all the cities developed specific strategies to contact and attract ”hard to reach” users, if necessary by combined outreach social service cooperating with police patrol or officers. A shared element in this was the provision of easily available contact and crisis centres that offered a range of social services, often incorporating needle dispensing, and in some cities, user rooms. Homelessness was addressed through a varied system of shelters and hostels, but the services presupposed appropriate behaviour and required the services’ premises to be respected by users. The dominant drug used in all the cities was heroin, and a high level use of injections often in public caused societal reactions and concerns in relation to epidemics of HIV and HCV. Overdoses and problematic overdose mortality both on scenes and in the neighbourhoods increased the need for interventions. This also made the available alternatives such as injection rooms and in particular low threshold methadone dispensing the obvious choices. Other drugs were also used, but the focus was on heroin.

One shared theme is that assertive social services seem to be a prerequisite of an effective response. Typically the cities have created active and assertive outreach services to contact and engage with drug using individuals on the street. This response was coordinated with or conjoint with police patrols or officers, in particular to prevent the development of drug scenes. The cities also provided a range of services such as night shelters and other housing opportunities, contact centres as a place to be during the day, and opportunity for cheap food, washing, needle dispensing etc. The service also served as a gateway to low threshold health services and maintenance programmes. But at the same time it was agreed that open drug scenes are destructive, and that the provision of services should not increase the attraction of the drug scenes and not be offered on scene.

The organization of health services in the cities varied but, again, some shared aspects were evident. One was an emphasis that services should also be available for the most behaviourally problematic users. The services tended not to operate with written referrals and timed appointments, but to be available on demand. Waiting lists and waiting times were reduced as far as possible and were regarded in principle as not acceptable. The substance use treatment service should be comprehensive and include low threshold methadone maintenance, rehabilitation oriented maintenance therapy (high threshold), crisis intervention, and detoxification within a longitudinal treatment perspective. The choice of abstinence oriented treatment and treatment in therapeutic communities was optional. Communication and coordination was strongly emphasized, either through coordination boards of high level commissioners. Information was to a large degree shared between controlling and helping offices,.

Some elements were found in some but not in all of the cities (Table 1). User rooms were integrated within the responses of Amsterdam, Frankfurt and Zürich. Respondents reported that these supported the social integration of users, particularly those without their own housing. The facilities were also seen as reducing public nuisance in local areas with a high prevalence of drug use. Heroin assisted treatment was a particular feature of the city responses of Zürich and Amsterdam while playing a more limited role in Frankfurt. Heroin assisted treatment was not seen as an essential response for the prevention of open drug scenes in any of the cities. Low threshold methadone treatment was emphasized in all cities, but a mobile service with dispensing in buses was only operative in Amsterdam and Lisbon. Slow release morphine (Substitol) was only used in Vienna where it was the main agonist medication, and was valued by doctors and users, but seen as problematic by the police. Reports suggested that slow release morphine was diverted and misused on a relatively large scale, and that more than half of the overdose deaths were found to be associated with this medication.

### Limitations

The findings relate to extremely complex events which took place in changing social, political and economic circumstances and often over relatively prolonged periods of time. The findings are largely based upon evidence given by individuals or groups with close access to events in each of the cities, but the expressed views are always subject to personal opinions about those events. In Amsterdam, we did not succeed in convening an inter-professional and interdisciplinary group and were dependent on information from and discussion with core researchers. In the other cities the use of multiple informants and multiple information sources was intended to reduce biases due to incomplete accounts or unrepresentative views, though this does not eliminate the problem of shared but inaccurate beliefs. Also, the biases or inaccuracies of the investigators were counterbalanced by the process of repeated feedback from respondents.

The focus on heroin as problematic drug might limit the usefulness for cities in which other types of drug are most strongly linked to open drug scenes. Use of crack cocaine has increased in Europe and has for many years been a major problem in some South American countries and some US city centers. On problematic aspect is the lack of evidence based low threshold services for these types of problems limiting the direct relevance when cocaine and amphetamine are the main drugs. However, the problems of policy disagreement and the need for coordinated interventions combining restricting and helping measures are in our opinion of clear relevance.

It should also be acknowledged that the investigation of complex social events such as the waxing and waning of open drug scenes is not amenable to traditional experimental designs, and “objective data” about the evolution of such events is extremely difficult (and sometimes impossible) to obtain. Provided that the limitations of the study methods are taken into account, we believe that the findings may be useful and informative.

## Conclusion

The cities in our study have all responded to open drug scenes through a combination of prevention, enforcement, harm reduction, and treatment measures as recommended by Bless [2]. They have also developed systems for active cooperation between police, health care and social services. They provided ready access to and availability of low threshold maintenance treatment, most often by methadone. As a consequence they appear to have effectively closed the existing open drug scenes and have continued to implement active measures to prevent any recurrence of the scenes. Underlying these measures has been a basic acceptance of drug users including those who have been unable or unwilling to stop the use of illegal drugs, but this was combined with a policy not to permit the continuation of destructive behaviour in terms of public nuisance. The cities established policies of “no tolerance” for public nuisance but nevertheless developed appeasement, and found approaches to “coexistence” between society and users of illegal substances. This helped to end unfruitful controversies between liberal and conservative ideologies and policies. The solutions are found in appropriate combinations of harm reduction and restrictive measures.

## Authors’ information

HW: Professor emeritus psychiatry and addiction medicine, University of Oslo, Norwegian Centre for Addiction Research and consultant at Oslo University Hospital, Clinic for psychiatry and addiction, Centre for substance abuse treatment. HW has been central in the development of opioid maintenance treatment and addiction treatment and research in Norway.

TC: Dr med, Professor of addiction medicine, SERAF, Norwegian Centre for Addiction Research, University of Oslo. Core interest: Norwegian cohort studies on opoid maintenance treatment, overdose mortality epidemiology and prevention and international alcohol dependency studies.

LG: PhD from Oslo University, Norwegian Centre for Addiction Research. Thesis on opioid maintenance treatment programs. Presently: Researcher at Norwegian Institute of Alcohol and Drug Research.

MG: Professor Kings College London, National Addiction Centre, UK. Wide international experience from different types of addiction research. Presently visiting professor at Norwegian Centre for Addiction Research, University of Oslo.

## Abbreviations

EMCDDA: European monitoring centre for drugs and drug addictions
SIP: Security, intervention, prevention
CDT: Commissions for dissuasion of drug addiction.
